# 1293. Efficacy of Doxycycline in Staphylococcal Prosthetic Joint Infection Treated with Debridement, Antibiotics and Implant Retention (DAIR) or Revision with Destination Spacer Placement

**DOI:** 10.1093/ofid/ofad500.1132

**Published:** 2023-11-27

**Authors:** Timothy Jang, Angela Hewlett, Nicolas W Cortes-Penfield

**Affiliations:** University of Nebraska Medical Center, Omaha, Nebraska; University of Nebraska Medical Center, Omaha, Nebraska; University of Nebraska Medical Center, Omaha, Nebraska

## Abstract

**Background:**

Prosthetic joint infection (PJI) is common, limb-threatening, and often warrants extended antibiotic durations. Intravenous (IV) glycopeptide, lipopeptide, and beta-lactam antibiotics have been the *de facto* US standard of care for staphylococcal PJI for decades, while the preponderance of (mostly European) published clinical data for PJI involves fluoroquinolone and rifampin combinations. However, these antibiotic strategies carry substantial adverse effects. In contrast, tetracyclines are less-studied for treatment of staphylococcal PJIs, but offer ease of administration and a favorable side effect profile.

**Methods:**

We retrospectively evaluated the characteristics and outcomes of patients with staphylococcal PJI treated with doxycycline from March 2019 to April 2021 who received some period of outpatient parenteral antibiotic therapy (OPAT) and were included in our medical center’s OPAT database. We included patients with hip, knee, or shoulder PJIs managed with either debridement, antibiotics and implant retention (DAIR) or revision to a permanent “destination” spacer who received doxycycline within the first 12 weeks of therapy. Diagnosis of staphylococcal PJI was based on the documentation of the treating ID team plus identification of staphylococci in surgical tissue or synovial fluid cultures. Our primary outcome of interest was treatment failure, defined as reoperation for infection in the affected joint within one year of initial surgery.

**Results:**

Of 24 patients switched to doxycycline after a mean 42 days, 17 underwent DAIR and 7 had destination spacers placed. 15 of 17 patients (88.2%) who underwent DAIR and 7 of 7 patients (100%) who received destination spacers remained free of treatment failure at one year; two patients had recurrent infections and none died.

**Conclusion:**

These findings suggest that doxycycline is a safe and efficacious option for treatment of PJI. A key limitation of this study is patient identification via the OPAT database, meaning patients with short IV lead-in therapy (i.e. switch to PO at discharge) were not included, and that the efficacy of doxycycline is likely exaggerated by immortal time.

**Disclosures:**

**Angela Hewlett, MD, MS**, Forcast Orthopedics: Advisor/Consultant|Mapp Biopharmaceutical: Grant/Research Support

